# Dancing in the sun: maize azimuthal canopy re-orientation for efficient light capture

**DOI:** 10.1093/plcell/koae026

**Published:** 2024-01-25

**Authors:** Min-Yao Jhu, Hokuto Nakayama

**Affiliations:** Assistant Features Editor, The Plant Cell, American Society of Plant Biologists; Crop Science Centre, Department of Plant Sciences, University of Cambridge, Cambridge CB30LE, UK; Department of Biological Sciences, The University of Tokyo, Tokyo 113-0033, Japan

Over the last seven decades, high-density planting has helped increase US maize grain yields, reaching more than 93,000 plants/ha in the mid-2010s (Assefa et al. 2018). However, high-density planting also brings challenges, such as increased shading, which can affect individual plant yield. These challenges prompt a closer look at canopy architecture, particularly leaf inclination angle and azimuthal canopy orientation. The leaf inclination angle to the horizontal plane is crucial for light penetration through the canopy and overall productivity. Historically, more erect leaves allow greater light penetration and have been linked to greater yield. Previous studies indicated that more hybrids with erect leaves were released after 1970 (Duvick et al. 2004). The changes in leaf inclination angle have been hypothesized to be related to higher plant density in use.

Beyond leaf inclination, azimuthal canopy orientation is also important for light interception, especially for densely planted crops. The azimuthal leaf angle refers to the distribution of leaves around a stem relative to the planting axis (i.e. row) as viewed from above, ranging from parallel to perpendicular to the row. The orientation of leaves around the stem relative to other leaves is also termed leaf phyllotaxy (e.g. alternate, opposite, or whorled). **Yan Zhou and co-workers** (Zhou et al. 2024) investigate azimuthal canopy re-orientation in maize, whereby plants adjust the azimuthal orientation of their leaves in response to environmental factors, especially higher versus lower planting densities. Their research reveals that several maize genotypes can re-orient their leaves toward interrow spaces to optimize light interception depending on the planting density. This intricate dance of the maize canopy reflects shade avoidance syndrome (SAS), a mechanism known to be triggered by changes in the red-to-far-red light ratios (R/FR) (Casal 2013). SAS involves responses such as erect leaf angles and stem elongation, which are well studied in *Arabidopsis* and tomatoes (Casal 2013). However, the link between SAS and azimuthal canopy re-orientation in maize remained unclear.

Zhou et al. conducted genome-wide association studies to identify genes associated with azimuthal canopy re-orientation. Their results revealed multiple trait-associated single-nucleotide polymorphisms linked to SAS-related genes, and mutants of some of these genes showed altered abilities in azimuthal canopy re-orientation. This observation suggests a link between SAS and canopy dynamics, affecting light interception and facilitating leaf re-orientation toward the interrow space. Unexpectedly, their study uncovered the involvement of *liguleless* (*lg*) genes, regulating ligule and auricle development (Lewis et al. 2014) in canopy orientation. *lg1* and *lg2* mutants showed altered azimuthal canopy orientations and negative phototropism, indicating their role in plant responses to neighboring plants. This discovery suggests an unknown aspect of *lg* gene function, intertwining with the broader light response and canopy re-orientation.

Zhou et al. proposed a model to explain how maize plants reorient their canopies in response to changes in plant density (see Fig.). The model suggests that reduced light levels due to increased shading lead to altered auxin synthesis and transport, ultimately influencing the azimuthal orientation of leaves. A reduced R/FR ratio in low light is sensed by phytochrome, triggering the synthesis of auxin, which is transported to the leaf base. Increased auxin levels stimulate *lg* gene expression and promote additional auxin synthesis and accumulation on the shaded side and subsequent cell elongation, gradually reorienting the leaf toward better-lit interrow spaces. Surprisingly, *lg* mutants lack canopy re-orientation and exhibit negative phototropism, a response to light in the wrong direction. Their study proposes future investigations into blue light perception, the transduction of light signals via auxin pathways, and the role of low auxin levels in negative phototropism in *lg* mutants. Investigating how *lg* genes regulate auxin synthesis and transport would provide a deeper understanding of the canopy re-orientation mechanism.

**Figure.**
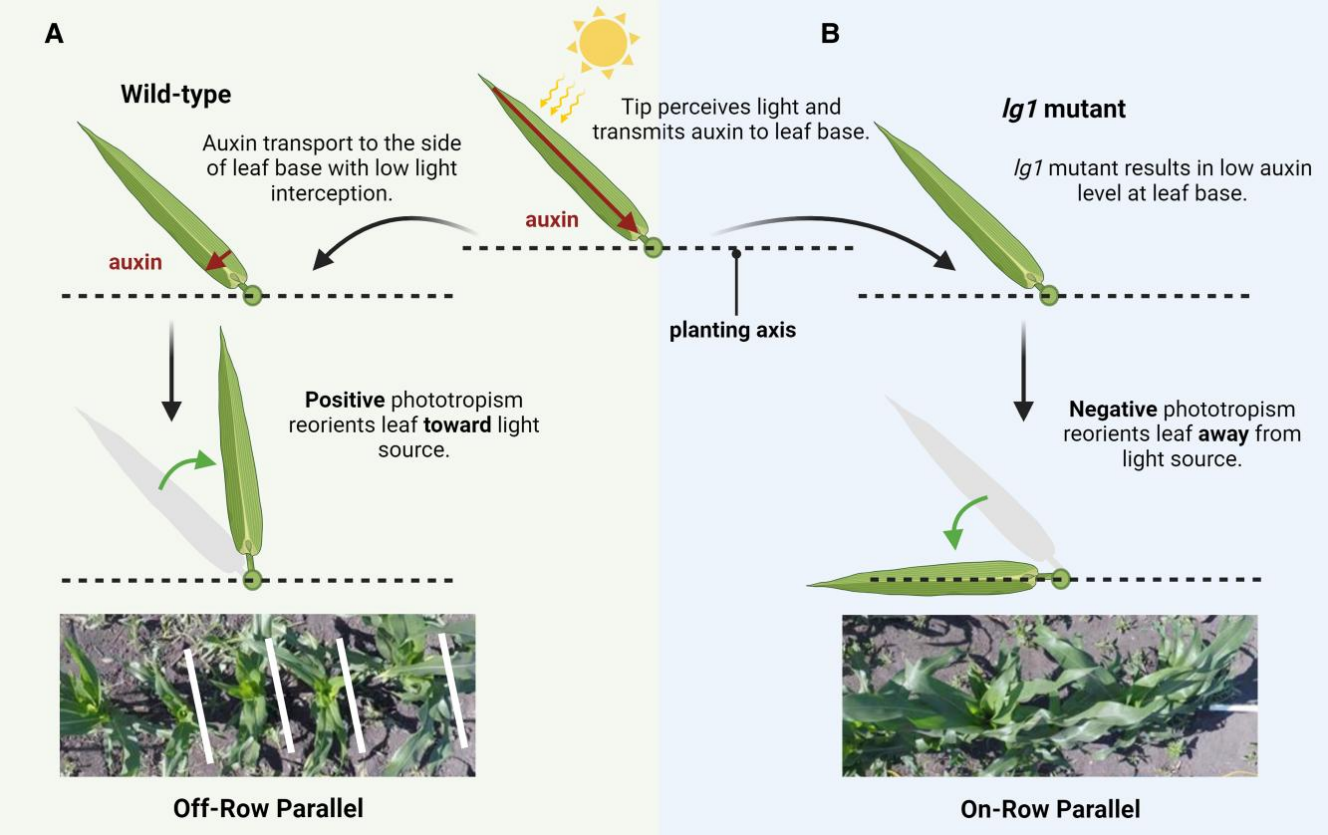
Proposed model of maize canopy re-orientation. **A)** In wild-type plants, auxin transport to the leaf base prompts additional auxin production, causing accumulation on the shaded side and leading to leaf orientation toward well-lit interrow spaces. **B)** In *lg1* mutants, *lg1* malfunction suppresses auxin-related genes at the leaf base, resulting in negative phototropism, with leaves turning away from light in the interrow space. The figure was recreated based on the model from Zhou et al. (2024), Figures 1 and 13, and was created using BioRender.com.

Although requiring further investigation, Zhou et al.'s study sheds light on an unexplored mechanism crucial for maize adaptation to increased plant densities. From plasticity in canopy re-orientation to *lg* genes’ multifaceted roles in canopy architecture and light responses, the research explains maize canopy re-orientation in response to varying environmental conditions—a dance guided by the sun's ever-changing tune. Extending similar studies to other plant species could uncover common or species-specific mechanisms of canopy re-orientation, contributing to broader agricultural applications.
